# A Comparative Analysis of Patient Demographics and Referral Trends Pre‐ and Post‐Intervention of the TIA Pathway: A Retrospective Observational Study

**DOI:** 10.1002/hsr2.72483

**Published:** 2026-05-04

**Authors:** Adenike Wejinya, Victoria Butler, Esther Mireku, Gail Gooding, Lawrence Achilles Nnyanzi

**Affiliations:** ^1^ School of Health & Life Sciences Teesside University Middlesbrough UK; ^2^ North Tees and Hartlepool NHS Foundation Trust Stockton on Tees UK; ^3^ Faculty of Public Health North East Regional CPD Advisor London UK; ^4^ SEETA University Mukono Uganda; ^5^ Faculty of Public Health Thammasat University Bangkok Thailand

**Keywords:** healthcare access, logistic regression, NHS Trust, referral pathways, socioeconomic status, transient ischaemic attack

## Abstract

**Background and Aims:**

Timely diagnosis of Transient Ischaemic Attack (TIA) is critical for stroke prevention. This study evaluated referral patterns, demographic predictors, and diagnostic outcomes before and after a service improvement intervention at North Tees and Hartlepool NHS Foundation Trust.

**Methods:**

A retrospective observational study was conducted using 2,167 patient records from 2021 to 2024. Demographic and referral data were analysed using IBM SPSS Statistics v27. Chi‐square analyses were used to assess associations between TIA diagnosis and age group, gender, local authority, and referral source (two‐sided, *p* < 0.05). A multivariable logistic regression was performed to identify independent predictors, with odds ratios (ORs) and 95% confidence intervals (CIs) used to assess the strength and precision of associations.

**Results:**

The TIA diagnosis rate was 37.8%. Older adults (≥ 65 years) accounted for 72.3% of TIA cases. Digital referrals (Email and WebICE) increased significantly post‐intervention, replacing GP and consultant routes. Consultant referrals, though rare, had the highest diagnostic yield (53.8%). Chi‐square analysis showed significant associations between TIA diagnosis and age group, gender, local authority, and referral source (*p* < 0.05). In regression analysis (*N* = 1959), increasing age and consultant referrals were associated with higher odds of diagnosis. Middle deprivation was associated with slightly lower odds (OR = 0.77, *p* = 0.05). Gender was not a statistically significant predictor (*p* = 0.16).

**Conclusion:**

TIA diagnosis is strongly associated with age and referral source. Service changes improved referral standardisation, with digital pathways becoming the dominant mode of referral. Clinical judgment appears to play an important role, as reflected in the higher diagnostic yield of consultant referrals. These findings highlight the importance of considering key demographic and system‐level factors in stroke prevention pathways.

## 1 Introduction

### 1.1 Background

Transient ischaemic attack (TIA), often referred to as ‘mini‐stroke’ is a brief episode of neurological dysfunction caused by temporary cerebral ischaemia without permanent tissue damage [1]. TIAs are considered important clinical warning events, with approximately 8.5% to 14.6% of patients experiencing an ischaemic stroke within 90 days of a TIA [2, 3].

The true prevalence of TIA remains unclear due to frequent underdiagnosis and misclassification, especially in emergency and outpatient settings [4, 5]. This uncertainty leads to inconsistent reporting and delays in secondary prevention [2, 6]. As a result, affecting both patient outcomes and the design of effective care pathways.

The National Institute for Health and Care Excellence [7] emphasises the need for rapid assessment and early intervention in specialist stroke services. Despite this, referral processes, patient education, and access to care remain inconsistent across the system. These issues are made worse by social and demographic factors. Research shows that socioeconomic status, age, gender, and location all influence access to stroke‐related services [8, 9].

In 2021, a baseline needs assessment was carried out at North Tees and Hartlepool NHS Foundation Trust. It identified several service‐level issues, including poor referral coordination and patient engagement, and gaps in health promotion. To address these, the Trust introduced three service improvements between 2022 and 2023:

i. A ‘Making Every Contact Count (MECC)’ questionnaire was introduced in TIA clinics. This is a public health approach that encourages healthcare professionals to use routine interactions to promote healthy behaviour. Patients received it before their consultant review. It provided signposting resources and acted as a prompt for MECC‐based discussions.
ii. Multidisciplinary patient education classes were reintroduced across Hartlepool and Stockton‐on‐Tees. Stroke nurses, physiotherapists, dietitians, and the Stroke Association delivered these sessions. They focused on secondary prevention, blood pressure and weight monitoring, and also involved carers and relatives.
iii. TIA awareness training was delivered to local authorities and care providers. This aimed to support early recognition and improve referral practices.

Despite these interventions, limited evidence exists on on how such changes affect access among different demographic or socioeconomic groups. There is also limited understanding of how referral pathways evolve over time [10, 11]. Most TIA research focuses on clinical outcomes and stroke risk. Very few studies explore pathway variation or equity in access [12]. This study aims to address that gap.

### 1.2 Study Aim

To undertake a comparative analysis of patient demographics and referral pathways within the TIA service, before and after service interventions, at North Tees and Hartlepool NHS Foundation Trust.

### 1.3 Objectives

- To analyse the demographic characteristics of patients across the pre‐ and post‐intervention periods.
- To investigate the relationship between TIA diagnosis and demographic factors (e.g., age, gender, local authority) and referral sources.
- To evaluate the variation in TIA diagnosis across deprivation levels and age groups.
- To assess changes in referral routes over time.
- To inform future service improvements in TIA management through targeted recommendations.

### 1.4 Methods

**Study Design:** This study used a retrospective comparative observational design. It analysed anonymised data from patients referred to the TIA clinic at North Tees and Hartlepool NHS Foundation Trust. The aim was to compare referral sources, patient demographics, and TIA diagnosis rates during two time periods: Pre‐intervention (2021–2022) and Post‐intervention (2023–2024). The study took place in the context of service changes made between 2022 and 2023. These included the introduction of the Making Every Contact Count (MECC) questionnaire, reinstatement of patient education sessions, and enhanced TIA awareness training. These interventions were not evaluated directly but provided background context.

**Study Periods**: The pre‐intervention period (2021–2022) before implementation of pathway changes. The post‐intervention period (2023–2024) was after the introduction of service interventions such as providing the Making Every Contact Count (MECC) questionnaire, reintroduction of patient education sessions, and TIA awareness training for local services.

**Setting and Data Source**: Data came from the Trust's TIA outpatient clinic database. This included routine clinical and demographic information. Each record contained: Age, gender, postcode, local authority, IMD (Index of Multiple Deprivation) category, referral source, Final diagnosis (TIA or no TIA). All data were anonymised before analysis.

**Population and Eligibility Criteria:** The study population included all adult patients (aged 18–100 years) referred to the TIA clinic between 2021 and 2024. Patients were eligible for inclusion if they:

- Resided within the defined local authorities served by the Trust;
- Were referred for suspected TIA or minor stroke and reviewed by a stroke consultant;
- Provided consent for data usage, or had consultee approval if lacking capacity.

### 1.5 Exclusion Criteria Included

- Age under 18 years;
- Non‐residency or planned relocation during the study period;
- Inability to provide informed consent and no available consultee.

Variables and Measures: The following demographic and referral variables were included in the analysis. Variables were categorised to enhance clinical interpretability and to align with NHS reporting conventions and service planning practices.

- Age (categorised into four groups: 18–24, 25–44, 45–64, and 65–100 years),
- Gender: Male and female;
- Local authority: Stockton‐on‐Tees, Hartlepool, and County Durham
- Socioeconomic status was assessed using the Index of Multiple Deprivation (IMD) 2019, based on patients' postcodes. Deciles were grouped into four categories for analysis: most deprived (deciles 1–2), middle deprivation (deciles 3–5), low deprivation (deciles 6–7), and least deprived (deciles 8–10) [13].
- Outcome: Final diagnosis as ‘TIA’ or ‘No TIA’
- Periods: Pre‐intervention and post‐intervention
- Referral Pathways used in this study (Table 1).

**Table 1:** Classification of referral pathways used in the study.

Referral type	Description	Classified as
Consultant	Referrals from hospital consultants submitted via email, WebICE or paper	Digital or non‐digital (based on method)
Email	Referrals submitted through secured NHS email	Digital
GP	Referrals initiated by genera; practitioners via phone or in person consultation	Non‐digital
Other	Includes referrals not falling into the above categories (such as unknown, unclassified)	Non‐digital
WebICE	Referrals submitted via the WebICE digital platform integrated EHR.	Digital

### 1.6 Data Analysis

Data were cleaned in Excel prior to analysis in IBM SPSS Statistics (version 27). Records with missing key variables or not meeting inclusion criteria (e.g., age < 18 years, residence outside the study area) were excluded, and analyses were conducted using a complete‐case approach. Descriptive statistics (means, medians, standard deviations, and percentages) summarised variable distributions. Associations between TIA diagnosis and categorical variables (age group, gender, IMD level, referral source) were assessed using Chi‐square tests, which were appropriate given the categorical data structure, independence of observations, and adequate expected cell counts [14].

Multivariable logistic regression was used to identify independent predictors of confirmed TIA diagnosis, with results reported as odds ratios (ORs) and 95% confidence intervals (CIs), following established methods [15]. All statistical tests were two‐sided, with a prespecified significance level of *p* < 0.05. Model fit was evaluated using the Hosmer–Lemeshow test [16].

Primary analyses were guided by the study objectives, while subgroup and stratified analyses were conducted for exploratory purposes in keeping with the retrospective design. Results were interpreted cautiously, particularly where *p* values were close to 0.05 or multiple comparisons were performed. Statistical reporting followed established recommendations for clinical research, with emphasis on effect sizes and appropriate interpretation of *p* values [17]. The study adhered to the STROBE reporting guidelines for observational studies (Appendix 1).

Ethical approval was obtained from Teesside University (Appendix 2) and the North Tees and Hartlepool NHS Foundation Trust. The study used anonymised secondary data and was deemed exempt from NHS REC review. Full details are provided in the Ethics Approval Statement.

## 2 Results

### 2.1 Demographic and Referral Trends Among TIA Patients Pre‐ and Post‐Intervention

**Study Population:** A total of 2,167 patient records were analysed. Of these, 819 (37.8%) were from the pre‐intervention period (2021–2022) and 1348 (62.2%) from the post‐intervention period (2023–2024). Data completeness was high, with valid entries ranging from 94.3% to 99.7% across key variables.

**Patient Age Distribution:** The average patient age remained consistent across both periods: Pre‐intervention: 69.17 ± 15.28 years; Post‐intervention: 68.58 ± 14.78 years. The overall mean age was 68.80 ± 14.97 years, with a median of 71 years and mode of 82 years. Most patients were in the older adult group (65+ years). Only 25 age values were missing, indicating strong data quality (Table 2).

**Table 2:** Summary statistics for patient age across pre‐ and post‐intervention periods.

Statistic	Pre‐intervention	Post‐intervention	Age 2021–2024
Valid (*N*)	798	1344	2142
Missing (*N*)	21	4	25
Mean	69.17	68.58	68.80
Median	71.00	70.00	71.00
Mode	78	76	82
Standard deviation	15.28	14.78	14.97
Variance	233.41	218.46	224.00
Minimum	19	20	19
Maximum	100	99	100

**Demographic Characteristics Across Time:** Demographic trends remained relatively stable (Table 3). Patients aged 65 and older represented 64.3% pre‐intervention and 63.5% post‐intervention. Gender distribution shifted slightly, with an increase in male patients from 46.2% to 48.9%. The majority of patients resided in Stockton‐on‐Tees (55.7%), followed by Hartlepool (30.7%) and County Durham (13.6%). IMD (Index of Multiple Deprivation) distribution showed consistency, with around one‐third of patients from the most deprived areas.

**Table 3:** Demographic characteristics, referral sources, and TIA diagnoses across study periods.

Demographic variable	Category	Pre‐intervention (%)	Post‐intervention (%)	Total (%)
Gender	Male	46.2	48.9	47.9
	Female	53.8	51.1	52.1
Age group	Young adult	0.5	0.1	0.3
	Adult	7.0	6.9	7.0
	Middle age	28.2	29.5	29.0
	Old age	64.3	63.5	63.8
Local authority	County Durham	14.3	13.2	13.6
	Stockton‐on‐Tees	56.3	55.4	55.7
	Hartlepool	29.3	31.4	30.7
Referral routes	Consultant	3.2	0.0	1.2
	Email	26.0	53.5	42.8
	GP	6.0	0.1	2.4
	WebICE	21.8	44.6	35.7
	Other	43.0	1.8	17.8
IMD	Most deprived	33.4	34.2	33.9
	Middle deprivation	23.8	24.9	24.5
	Low deprivation	14.4	13.0	13.5
	Least deprivation	28.4	27.9	28.1
TIA diagnosis	No TIA	64.1	61.1	62.2
	TIA	35.9	38.9	37.8

*Note: n* = 819 (pre), *n* = 1348 (post) (*n* = number of patients or cases before and after intervention).

**Referral Pathways:** A notable shift was observed in referral methods post‐intervention (Table 3). Email referrals increased from 26.0% to 53.5%. WebICE referrals rose from 21.8% to 44.6%. GP referrals dropped sharply from 6.0% to 0.1%. Consultant referrals fell from 3.2% to 0.0%. The ‘Other’ category reduced from 43.0% to 1.8%. This reflects a significant transition to digital referral pathways and increased standardisation of referral processes.

**TIA Diagnosis Trends**: Overall TIA diagnosis rate increased slightly from 35.9% pre‐intervention to 38.9% post‐intervention (Table 3). Diagnosis rates by gender showed lower proportions among women, though differences were not statistically significant. IMD category distributions remained stable, with no evidence of significant change across deprivation levels (Table 3). Chi‐square tests confirmed that only referral routes changed significantly over time (X² = 752.82, df = 4, *p* < 0.001), while gender, age group, local authority, deprivation level, and TIA diagnosis status showed no statistically significant variation across the two periods (Table 4). Only referral source changed significantly over time (*p* < 0.001), confirming the impact of the intervention on referral standardisation.

**Table 4:** Chi‐Square analysis of demographics, referral pathways, and TIA diagnosis by time.

Variables	X^2^	df	*p* value	Interpretation
Gender	1.53	1	0.22	Not significant
Age	0.75	3	0.86	Not significant
Local authority	1.14	2	0.57	Not significant
Referral routes	752.82	4	< 0.001	Significant
IMD	1.03	3	0.80	Not significant
TIA diagnosis	1.89	1	0.17	Not significant

*Note:* Where X^2^ = Chi‐square value; df = Degree of Freedom; *p* value = Probability value.

### 2.2 Association Between TIA Diagnosis and Patient Demographics and Referral Pathways

In Table 5, older adults (65+ years) had the highest diagnosis rate (43.2%), followed by middle‐aged patients (32.4%). Diagnosis was least likely among younger adults. Males had a higher proportion of confirmed TIA cases (40.4%) compared to females (35.6%). Patients from Stockton‐on‐Tees had the highest TIA diagnosis rate (41.0%), compared to Hartlepool (35.6%) and County Durham (34.6%) (Table 5). This may reflect population differences rather than underlying clinical variation. Consultant referrals yielded the highest diagnosis rate (53.8%) but represented a small group (n = 26). Referrals via Email and WebICE had similar rates ( ~ 39%), while GP and Other referral methods were associated with lower diagnosis rates (34.0% and 32.0%, respectively) (Table 5). Patients from less deprived areas were more likely to receive a TIA diagnosis: Least deprived: 42.0%; Low deprivation: 41.7%; Most deprived: 37.2%; Middle deprivation: 35.6% (Table 5). While the gradient suggests a socioeconomic pattern, statistical tests were used to confirm significance.

**Table 5:** Distribution of TIA diagnoses by demographic and service‐related factors.

Variables	Category	No TIA (*N*)	TIA (*N*)	Total cases (*N*)	TIA diagnosed (%)
Gender	Male	610	414	1024	40.4
	Female	718	397	1115	35.6
Age group	Young adult	6	0	6	0
	Adult	125	24	149	16.1
	Middle age	420	201	621	32.4
	Old age	776	590	1366	43.2
Local authority	County Durham	183	97	280	34.6
	Stockton‐on‐Tees	676	469	1145	41.0
	Hartlepool	406	224	630	35.6
Level of deprivation (IMD)	Most deprived	435	258	693	37.2
	Middle deprivation	322	178	500	35.6
	Low deprivation	161	115	276	41.7
	Least deprivation	333	241	574	42.0
Referral routes	Consultant	12	14	26	53.8
	Email	550	351	901	39.0
	GP	33	17	50	34.0
	WebICE	458	294	752	39.1
	Other	251	118	369	32.0

Table 6 shows the results of the chi‐square tests. Statistically significant relationships were found between TIA diagnosis and age group (X² = 57.95, *p* < 0.001), gender (X² = 5.28, *p* = 0.02), local authority (X² = 7.00, *p* = 0.03), and referral route (X² = 9.52, *p* = 0.05). These findings suggest that these factors were significantly associated with the likelihood of a confirmed TIA diagnosis. In contrast, the Index of Multiple Deprivation (IMD) showed no statistically significant association with TIA diagnosis (*p* = 0.10), indicating that deprivation level did not significantly influence diagnosis rates in this dataset.

**Table 6:** Statistical analysis of TIA diagnosis with demographic factors and referral routes.

Variables	X^2^	df	*p* value	Statistical result
Age group	57.95	3	< 0.001	Significant
Gender	5.28	1	0.02	Significant
Local authority	7.00	2	0.03	Significant
Level of deprivation (IMD)	6.29	3	0.10	Not Significant
Referral routes	9.52	4	0.05	Significant

*Note:* Where X^2^ = Chi‐square value; df = Degree of Freedom; *p* value = probability value.

### 2.3 Variation in TIA Diagnosis by Age and Deprivation Levels

**Figure 1:**
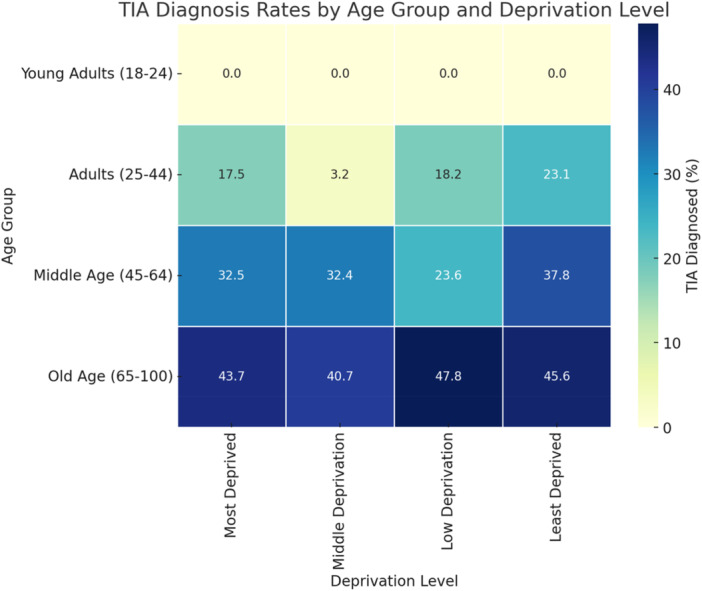
TIA diagnosis by age groups and deprivation levels.

In this study, a total of 2,038 patient records (94.0%) had complete data for age group, Index of Multiple Deprivation (IMD) category, and TIA diagnosis status. In Figure 1, the likelihood of TIA diagnosis increased significantly with age. Older adults (65–100 years) accounted for 72.3% of all confirmed TIA cases, followed by middle‐aged adults (45–64 years) at 24.9%, and adults (25–44 years) at 2.8%. No TIA diagnoses were recorded among young adults aged 18–24 years, highlighting the age‐dependent nature of TIA presentation (Figure 1).

Across IMD categories, the TIA diagnosis rate was highest among patients from the least deprived areas (42.0%), followed by those from low (41.7%), most deprived (37.2%), and middle deprivation (35.6%) areas. Although the variation was modest, the pattern suggests slightly higher diagnosis frequencies in less deprived communities (Figure 1). Chi‐square analysis revealed a significant association between TIA diagnosis, age group, and deprivation level (*p* < 0.001). Although TIA rates were slightly higher in less deprived areas, the strongest statistical associations were observed in the most and middle deprived groups, suggesting underlying differences in risk factors or healthcare access. These findings suggest variation in TIA diagnosis across age groups within deprivation categories and are summarised in Table 7.

**Table 7:** Chi‐square test results for TIA diagnosis across age groups and deprivation categories.

Variables	X^2^	df	*p* value	Statistical result
Most deprived	20.37	3	< 0.001	Significant
Middle deprivation	19.47	3	< 0.001	Significant
Low deprivation	13.12	2	0.001	Significant
Least deprivation	9.63	3	0.02	Significant (weaker)
Total	58.76	3	< 0.001	Highly Significant

*Note:* Where X^2^ = Chi‐square value; df = Degree of Freedom; *p* value = probability value.

### 2.4 Comparative Trends in TIA Referrals and Diagnoses Across Intervention Periods

**Figure 2:**
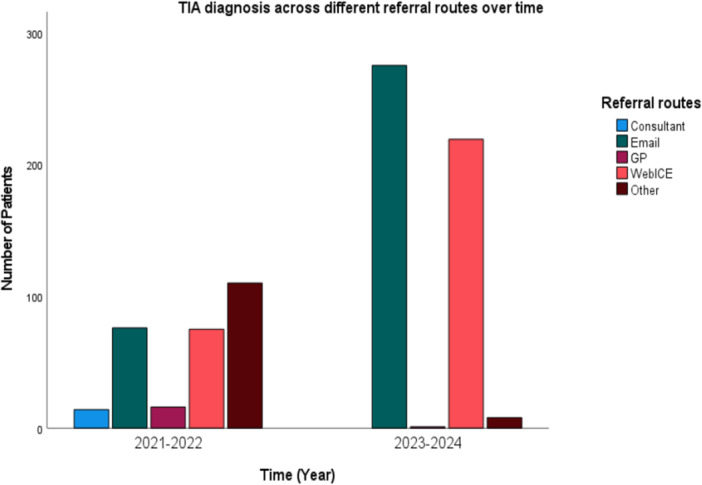
TIA diagnosis rate and referral routes pattern over time.

Of the 2,167 patient records analysed, 2,098 (96.8%) had valid data on referral route, intervention periods, and TIA diagnosis. In Figure 2, a shift in referral practices was observed between the pre‐ and post‐intervention periods. In pre‐intervention (2021–2022), referrals were distributed across several sources, with “other” accounting for 37.8%, Email 26.1%, and WebICE 25.8%. Consultant and GP referrals were also present, at 4.8% and 5.5%, respectively. While in the post‐intervention (2023‐2024), digital referrals dominated: Email increased to 54.7% and WebICE to 43.5%, while consultant and GP referrals dropped to 0% and 0.2%. The use of “other” routes declined to 1.6% (Figure 2).

Table 8 shows that chi‐square analysis confirmed a statistically significant changes for both TIA‐diagnosed and non‐diagnosed patients. The association between referral route and intervention period remained strong across all subgroups (X² = 744.68, df = 4, *p* < 0.001), supporting the hypothesis that referral patterns varied significantly over time. These findings suggest a successful transition to streamlined, electronic referral pathways following service‐level changes.

**Table 8:** Chi‐Square analysis of TIA diagnosis by referral route across pre‐ and post‐intervention periods.

TIA Status	X^2^	df	*p* value	Statistical result
No TIA Diagnosis	483.63	4	< 0.001	Significant
TIA Diagnosis	260.74	4	< 0.001	Significant
Total	744.68	4	< 0.001	Significant

*Note:* Where X^2^ = Chi‐square value; df = Degree of Freedom; *p* value = probability value.

### 2.5 Predictors of TIA Diagnosis: Multivariable Logistic Regression Analysis

A multivariable logistic regression model was conducted to assess whether demographic and referral characteristics were independently associated with a confirmed TIA diagnosis. The model was statistically significant (X²(11) = 79.80, *p* < 0.001) and showed a good fit based on the Hosmer‐Lemeshow test (*p* = 0.98). Table 9 presents the odds ratios (OR), confidence intervals, and significance levels for the predictors. In this analysis, the reference categories were chosen as follows: referral route = other; age group = 65 + , deprivation level =most deprived, and gender = female. These categories were selected based on clinical relevance or size of the subgroup, allowing for meaningful comparison across groups.

**Table 9:** Multivariable logistic regression analysis predicting confirmed TIA diagnosis (*N* = 1,959).

Predictor		Odds ratio (OR)	95% Cl	*p* value
Gender	Male	1.14	0.95–1.38	0.16
Age	25–44	0.24	0.15–0.38	< 0.001
	45–64	0.6	0.48–0.74	< 0.001
IMD	Middle deprivation	0.77	0.60–0.99	0.05
	Low deprivation	0.9	0.67–1.22	0.51
	Least deprivation	1.0	0.90–1.22	0.24
Referral sources	Consultant	2.57	1.07–6.19	0.04
	Email	1.26	0.95–1.66	0.10
	GP	1.1	0.57–2.11	0.78
	WebICE	1.27	0.96–1.69	0.09

*Note:* Reference categories: Referral = Other; Age = 65+; IMD = Most deprived; Gender = Female.

Referral source remained a key predictor. Patients referred by consultants had significantly higher odds of TIA diagnosis compared to those referred via “other” methods (OR = 2.57, 95% CI [1.07, 6.19], *p* = 0.04). Age was also a significant factor. Patients aged 45‐64 had lower odds of diagnosis (OR = 0.60, *p* < 0.001), as did those aged 25–44 (OR = 0.24, *p* < 0.001), compared to those aged 65 and above. Patients from areas with middle deprivation had lower odds of TIA diagnosis compared to those in the most deprived group (OR = 0.77, 95% CI [0.60, 0.99], *p* = 0.05). Gender was not a statistically significant predictor (OR = 1.14, *p* = 0.16). Overall, the model correctly classified 61.1% of cases; however, the sensitivity for detecting true TIA cases was low (10.4%), suggesting limited predictive accuracy using demographic and referral data alone.

## 3 Discussion

### 3.1 Demographic and Referral Trends Among TIA Patients Pre‐ and Post‐Intervention

This study reaffirms age as a major determinant of TIA diagnosis, with individuals aged 65+ years comprising over 72% of confirmed cases. This aligns with existing evidence that cerebrovascular risk increases with age due to accumulated vascular pathology [1, 18]. The prevalence of risk factors such as hypertension and atrial fibrillation also rises in older adults, increasing the baseline risk of transient or permanent ischaemic events [19]. Graham et al. [20] similarly demonstrated that older adults and males are more likely to be diagnosed with TIA or minor stroke, likely due to higher vascular risk burden and diagnostic pathway interactions.

A modest gender disparity was observed, with TIA diagnosis more common in males (40.4%) than females (35.6%). This mirrors findings from national stroke registries [21] and supports existing concerns about diagnostic delays in women, who may present with atypical or less recognised symptoms [22]. Underdiagnosis in women has been documented even when classical symptoms are present, suggesting potential diagnostic biases [23]. These findings support calls for gender‐sensitive diagnostic training and awareness.

Referral pathways changed significantly post‐intervention. Digital methods (Email, WebICE) became dominant, while non‐standard routes'other' decreased. This reflects successful implementation of pathway standardisation efforts and may support more standardised referral processes [24]. Digital transformations are particularly important in TIA services, where timely assessment is critical to prevent early stroke recurrence [25].

### 3.2 Predictors of TIA Diagnosis: Multivariable Insights

The logistic regression model identified consultant referral as a significant independent predictor of TIA diagnosis (OR = 2.57, *p* = 0.04). This suggests that hospital‐based referrals may reflect more clearly defined or severe symptomatology. This study is consistent with evidence that secondary care referrals are often triggered by higher clinical suspicion and urgency [26].

Age was also a strong predictor. Patients aged 25–44 and 45–64 had significantly lower odds of receiving a TIA diagnosis compared to those aged 65 + . This supports the well‐established association between increasing age and cerebrovascular risk [1, 8]. This age‐related diagnostic trend reflects both the biological accumulation of vascular risk and the greater likelihood of TIA‐mimicking conditions in younger populations.

An interesting finding was the reduced odds of TIA diagnosis among patients from middle deprivation areas compared to those in the most deprived group (OR = 0.77, *p* = 0.05). While this association was modest, it could reflect complex patterns in health‐seeking behaviour, access to care, or variation in clinical presentation across socioeconomic strata. Previous research has shown that health inequalities do not always follow linear deprivation gradients and may be shaped by local service structures, access pathways, and public health awareness [27]. In particular, the Marmot Review emphasises that place‐based factors, such as service design and community engagement, play a critical role in mediating health outcomes across socioeconomic groups.

Gender was not a statistically significant predictor of TIA diagnosis in the regression model (OR = 1.14, *p* = 0.16), though males had slightly higher odds. This aligns with findings from Asdaghi et al. [15], who reported no significant gender difference after adjusting for clinical variables. In contrast, studies by Bushnell et al. [28] found that women were less likely to be diagnosed with TIA, often due to atypical presentations and diagnostic biases. The lack of significance here may reflect the impact of standardised referral protocols and the absence of clinical symptom data in the model.

### 3.3 Association Between TIA Diagnosis and Patient Demographics and Referral Pathways

This study revealed statistically significant associations between TIA diagnosis and patient demographics including age, gender, local authority, and referral source. Older adults (≥ 65 years) had the highest proportion of confirmed TIA diagnoses (43.2%), consistent with established evidence linking increasing age with higher cerebrovascular risk due to cumulative vascular pathology and comorbidities such as atrial fibrillation and hypertension [1, 18].

Male patients were more likely than females to receive a TIA diagnosis (40.4% vs. 35.6%, *p* = 0.02). This aligns with findings from population‐based studies showing sex differences in both symptom presentation and clinical response [22, 23]. Although the logistic regression model did not find gender to be an independent predictor (*p* = 0.16), the descriptive and bivariate results suggest subtle sex‐based diagnostic variation which may reflect biases or differences in health‐seeking behavior.

The consultant referrals had the highest proportion of confirmed TIA diagnoses (53.8%). This support prior studies by Rothwell et al. [26] that secondary care referrals often reflect more clinically urgent or clearly defined presentations. This underscores the continued importance of clinical acumen in enhancing diagnostic accuracy. In contrast, digital referrals (Email and WebICE) accounted for the majority of referrals post‐intervention and achieved moderate diagnostic performance (~39%). This suggests a more standardised referral process, with slightly lower diagnostic precision compared to consultant input. These findings are consistent with those of Asthana et al. [9], who demonstrated that digital referral systems can improve triage process and reduce service fragmentation within healthcare pathways.

### 3.4 Variation in TIA Diagnosis by Age and Deprivation Levels

There was a clear and statistically significant variation in TIA diagnosis across age groups (*p* < 0.001). Older adults (65+) represented 72.3% of all confirmed TIA cases, consistent with existing literature linking cerebrovascular risk to aging [8, 19]. Conversely, no diagnoses were made among patients aged 18–24 years, further highlighting the age‐dependent nature of TIA presentations.

Socioeconomic deprivation showed more complex patterns. Although patients from the least deprived areas had the highest proportion of confirmed TIA diagnoses (42.0%), followed by those from low (41.7%), most deprived (37.2%), and middle deprivation (35.6%) areas, these differences were not statistically significant at the group level (*p* = 0.10). This contrasts with prior studies which have demonstrated that patients from more deprived backgrounds often face higher stroke and TIA risks, driven by greater exposure to modifiable risk factors and structural healthcare barriers [29, 30].

One possible explanation for this finding is that features of the pathway may influence access patterns. However, this cannot be confirmed from the present data. Nonetheless, chi‐square subgroup analyses showed that the most and middle deprived categories contributed significantly to variation in diagnosis (*p* < 0.001). This suggests nuanced differences in risk exposure or healthcare interaction that warrant further qualitative investigation.

### 3.5 Comparative Trends in TIA Referrals and Diagnoses Across Intervention Periods

The intervention period was marked by statistically significant shift in referral practices (X² = 744.68, *p* < 0.001). Digital referral methods (Email and WebICE) replaced non‐digital methods (Consultant and GP). Email referrals increasing from 26.1% to 54.7% and WebICE from 25.8% to 43.5%. In contrast, consultant and GP referrals fell to near‐zero levels after the intervention. These changes reflect successful implementation of pathway reforms intended to streamline access and improve referral quality.

This shift is in line with NHS‐wide digitalisation initiatives aimed at modernising care delivery and supporting referral standardisation [31]. The improved referral standardisation observed in this study parallels previous findings demonstrating that electronic systems can enhance care coordination in time‐sensitive conditions like TIA [9, 32]. Despite their decline in frequency, consultant referrals retained the highest diagnostic precision, suggesting that digital systems should ideally incorporate clinical decision support or specialist review features to maintain diagnostic quality [33].

### 3.6 Study Strengths and Limitations

This study has several strengths. It utilised a large, real‐world dataset of over 2,100 patients referred to a UK secondary care TIA clinic (North Tees and Hartlepool NHS Foundation Trust), enhancing the external validity and relevance of the findings. The use of routinely collected data minimised recall bias and allowed the examination of naturally occurring referral and diagnostic patterns. Comparative analysis across pre‐ and post‐intervention periods enabled assessment of system‐level changes over time. In addition, subgroup analysis by age and deprivation provided important insights into healthcare equity and potential disparities in TIA diagnosis.

However, some limitations should be noted. The study relied on secondary data, which included missing values for key variables such as Index of Multiple Deprivation (IMD), local authority, and referral source. Although data completeness was high, this may have introduced minor bias and reduced statistical power. The evaluation did not assess the direct impact of specific service interventions due to the absence of detailed data on implementation. Differences in population size across local authorities were not accounted for, which may limit the generalisability of area‐based comparisons. The study did not include process‐based measures, limiting the ability to evaluate referral efficiency. Non‐significant findings related to deprivation should be interpreted cautiously due to the potential for residual confounding. As an observational study, causal relationships cannot be established. Finally, the logistic regression model was based on non‐clinical variables; clinical details such as symptom presentation and comorbidities were not included, limiting the predictive strength of the model.

## 4 Conclusion

This study demonstrates that age and referral source are key factors associated with TIA diagnosis. Individuals aged ≥ 65 years accounted for the majority of confirmed TIA cases. A modest gender difference was observed, with higher diagnosis rates among men. No significant association was found between TIA diagnosis and deprivation. However, this does not exclude the possibility of residual confounding or underlying disparities across socioeconomic groups. Digital referrals (Email and WebICE) became the dominant methods, contributing to greater referral standardisation. Continued pathway review and targeted outreach may help sustain diagnostic quality and improve service delivery.

## 5 Recommendations

- Future service evaluations should incorporate clinical variables (such as symptoms, comorbidities) to enhance diagnostic modelling and identify hidden disparities not captured by demographic data alone.
- Training for healthcare providers should include gender‐sensitive approaches to reduce potential underdiagnosis of TIA in women, even when classical symptoms are present.
- Digital referral systems such as Email and WebICE should be sustained and optimised, as they improved post‐intervention standardisation of referral processes.
- Despite being rare, consultant referrals had the highest diagnostic accuracy and should be integrated into complex case triage or supported through clinical decision tools.
- Further research into sex‐specific symptom patterns in TIA is also needed to reduce diagnostic disparities affecting women.

## Author Contributions

**Adenike Wejinya:** writing – original draft, methodology, validation, visualization, writing – review and editing, software, formal analysis, resources, project administration, investigation. **Victoria Butler:** conceptualization, investigation, supervision, writing – original draft. **Esther Mireku:** conceptualization, validation, supervision. **Gail Gooding:** data curation. **Lawrence Achilles Nnyanzi:** methodology, supervision, validation.

## Funding

This study was conducted as part of a postgraduate dissertation and received no external funding. No specific grant from any funding agency in the public, commercial, or not‐for‐profit sectors was received for this research.

## Ethics Statement

This study received ethical approval from Teesside University (Appendix 2) and the North Tees and Hartlepool NHS Foundation Trust Research Office. The study used anonymised secondary data; informed consent was waived in accordance with institutional and NHS governance requirements.

## Conflicts of Interest

The authors declare no conflicts of interest.

## Transparency Statement

The lead author ADENIKE WEJINYA, LAWRENCE ACHILLES NNYANZI affirms that this manuscript is an honest, accurate, and transparent account of the study being reported; that no important aspects of the study have been omitted; and that any discrepancies from the study as planned (and, if relevant, registered) have been explained.

## Data Availability

The data that support the findings of this study are available on request from the corresponding author. The data are not publicly available due to privacy or ethical restrictions.

## Appendix 1

**Table:**

Item Number	Section	Recommendation	Addressed in manuscript
1	Title and abstract	Indicate study design with commonly used terms in the title or abstract	Yes, in title page and abstract
2	Introduction	Explain scientific background and rationale for investigation.	Yes
3	Objectives	State specific objectives and hypotheses	Yes, in introduction and study aim section
4	Study design	Present key elements of study design early in the paper.	Yes, Methods section
5	Setting	Describe setting, locations, and dates of recruitment.	Yes, Methods
6	Participants	Give eligibility criteria and sources of participants.	Yes
7	Variables	Clearly define outcomes, exposures, predictors.	Yes, Variables section
8	Data sources/measurement	Provide sources of data and assessment methods.	Yes
9	Bias	Describe efforts to address bias.	Yes, Limitations section
10	Study size	Explain how study size was arrived at.	Yes, *N* = 2,167 in Methods
11	Quantitative variables	Explain handling of quantitative variables.	Yes
12	Statistical methods	Describe statistical methods and any adjustments.	Yes, method section
13	Participants	Report numbers at each stage of study.	Yes, throughout Results
14	Descriptive data	Give characteristics of study participants.	Yes, Tables 1, 2, 3, 4
15	Outcome data	Report numbers of outcome events.	Yes
16	Main results	Give unadjusted estimates and precision.	Yes, chi‐square results and tables
17	Other analysis	Report subgroup analyses and interactions.	Yes, results sections with IMD and age
18	Key results	Summarise key results with reference to objectives.	Yes, in discussion
19	Limitations	Discuss study limitations and bias.	Yes, Limitations section
20	Interpretation	Provide cautious interpretation of results.	Yes
21	Generalisability	Discuss external validity of results.	Yes, limitations and discussion
22	Funding	Give funding source and role of funders.	Yes, funding information section
